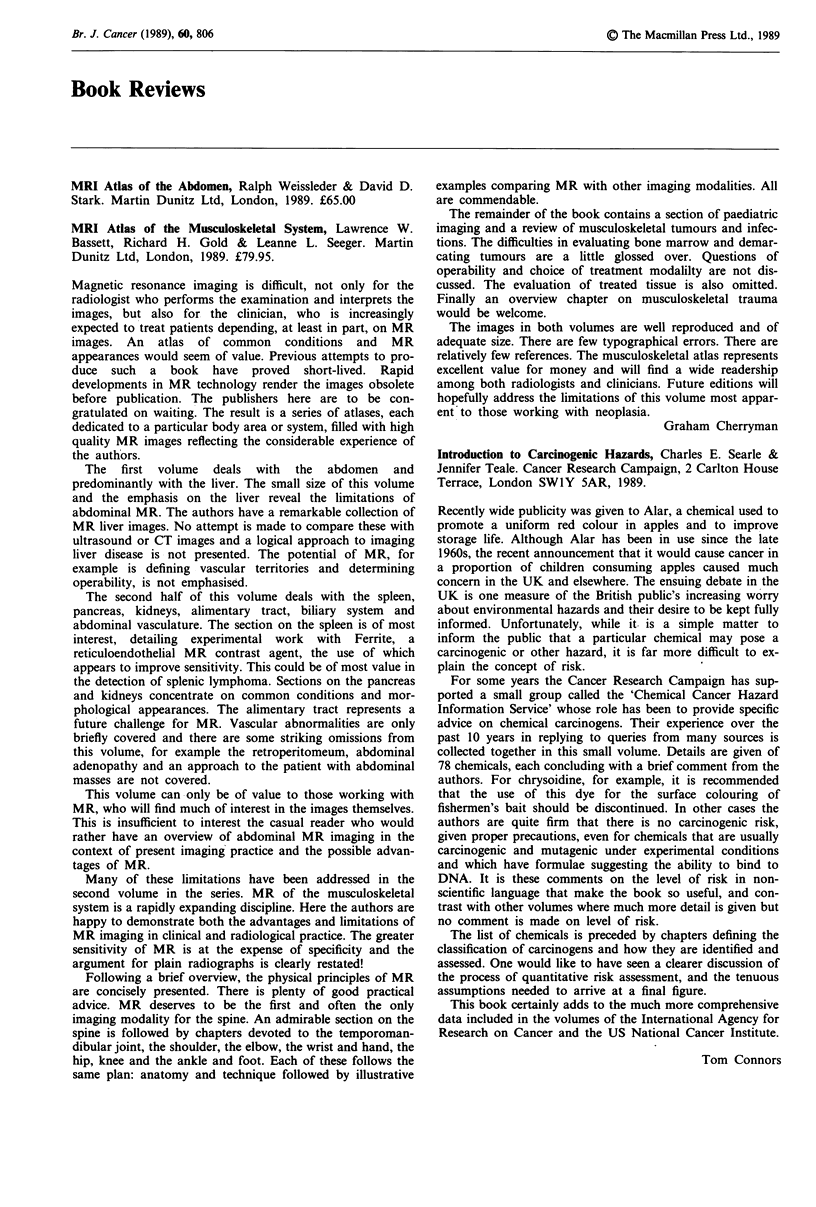# MRI Atlas of the Abdomen

**Published:** 1989-11

**Authors:** Graham Cherryman


 © The Macmillan Press Ltd., 1989

### Book Reviews

**MRI Atlas of the Abdomen,** Ralph Weissleder & David D. Stark. Martin Dunitz Ltd, London, 1989. £65.00

**MRI Atlas of the Musculoskeletal System,** Lawrence W. Bassett, Richard H. Gold & Leanne L. Seeger. Martin Dunitz Ltd, London, 1989. £79.95.

Magnetic resonance imaging is difficult, not only for the radiologist who performs the examination and interprets the images, but also for the clinician, who is increasingly expected to treat patients depending, at least in part, on MR images. An atlas of common conditions and MR appearances would seem of value. Previous attempts to produce such a book have proved short-lived. Rapid developments in MR technology render the images obsolete before publication. The publishers here are to be congratulated on waiting. The result is a series of atlases, each dedicated to a particular body area or system, filled with high quality MR images reflecting the considerable experience of the authors.

The first volume deals with the abdomen and predominantly with the liver. The small size of this volume and the emphasis on the liver reveal the limitations of abdominal MR. The authors have a remarkable collection of MR liver images. No attempt is made to compare these with ultrasound or CT images and a logical approach to imaging liver disease is not presented. The potential of MR, for example is defining vascular territories and determining operability, is not emphasised.

The second half of this volume deals with the spleen, pancreas, kidneys, alimentary tract, biliary system and abdominal vasculature. The section on the spleen is of most interest, detailing experimental work with Ferrite, a reticuloendothelial MR contrast agent, the use of which appears to improve sensitivity. This could be of most value in the detection of splenic lymphoma. Sections on the pancreas and kidneys concentrate on common conditions and morphological appearances. The alimentary tract represents a future challenge for MR. Vascular abnormalities are only briefly covered and there are some striking omissions from this volume, for example the retroperitomeum, abdominal adenopathy and an approach to the patient with abdominal masses are not covered.

This volume can only be of value to those working with MR, who will find much of interest in the images themselves. This is insufficient to interest the casual reader who would rather have an overview of abdominal MR imaging in the context of present imaging practice and the possible advantages of MR.

Many of these limitations have been addressed in the second volume in the series. MR of the musculoskeletal system is a rapidly expanding discipline. Here the authors are happy to demonstrate both the advantages and limitations of MR imaging in clinical and radiological practice. The greater sensitivity of MR is at the expense of specificity and the argument for plain radiographs is clearly restated!

Following a brief overview, the physical principles of MR are concisely presented. There is plenty of good practical advice. MR deserves to be the first and often the only imaging modality for the spine. An admirable section on the spine is followed by chapters devoted to the temporomandibular joint, the shoulder, the elbow, the wrist and hand, the hip, knee and the ankle and foot. Each of these follows the same plan: anatomy and technique followed by illustrative examples comparing MR with other imaging modalities. All are commendable.

The remainder of the book contains a section of paediatric imaging and a review of musculoskeletal tumours and infections. The difficulties in evaluating bone marrow and demarcating tumours are a little glossed over. Questions of operability and choice of treatment modalilty are not discussed. The evaluation of treated tissue is also omitted. Finally an overview chapter on musculoskeletal trauma would be welcome.

The images in both volumes are well reproduced and of adequate size. There are few typographical errors. There are relatively few references. The musculoskeletal atlas represents excellent value for money and will find a wide readership among both radiologists and clinicians. Future editions will hopefully address the limitations of this volume most apparent to those working with neoplasia.

Graham Cherryman